# A Smart Spatial Routing and Accessibility Analysis System for EMS Using Catchment Areas of Voronoi Spatial Model and Time-Based Dijkstra’s Routing Algorithm

**DOI:** 10.3390/ijerph20031808

**Published:** 2023-01-18

**Authors:** Abdullah Alamri

**Affiliations:** College of Computer Science and Engineering, University of Jeddah, Jeddah 23890, Saudi Arabia; amalamri@uj.edu.sa

**Keywords:** spatial database, spatial analysis, dijkstra’s algorithm, voronoi diagram, public health emergency

## Abstract

The concept of a catchment area is often used to establish equitable access to essential services such as ambulance emergency medical services. In a time-sensitive environment, taking the wrong decision when there is a need for a short travel time can have serious consequences. In ambulance management, a mistaken dispatch which may result in the late arrival of an ambulance can lead to a life-and-death situation. In addition, finding the optimal route to reach the destination within a minimum amount of time is a significant problem. A spatial routing analysis based on travel times within the emergency services catchment area can quickly find the best routes to emergency points and may overcome this problem. In this study, a smart spatial routing and accessibility analysis system is proposed for EMS using catchment areas of the Voronoi spatial model and time-based Dijkstra’s routing algorithm (TDRA) to support the route analysis of emergencies and to facilitate the dispatch of appropriate units that are able to respond within a reasonable time frame. Our simulation shows that the system can successfully predict and determine the nearest candidate ambulance unit within the catchment area and candidate ambulance services in the adjacent catchment area that has a minimum travel time to the demand point taking TDRA construction into account.

## 1. Introduction

Recently, emergency response times have attracted great attention from urban managers and researchers as they are considered the main factor for determining the success of pre-hospital care [1,2,3]. Decisions on the location and dispatching of an emergency unit are both critical and difficult for any emergency medical service (EMS). A time-based prediction is required for some time-sensitive services, such as ambulances, in order to send the appropriate unit in response to an emergency scenario since a mistaken assignment might result in a life-or-death situation [4].

Most countries have developed some type of emergency medical service in order to provide first assistance in emergencies. These services are administered by operators who must determine which unit is closest to the emergency area [5]. Their operating environment is characterized by uncertainty since they do not know the exact distance between each unit and the emergency site. When an emergency occurs, it is imperative that accurate data are available and that prompt action is taken. An effective emergency response mechanism can be integrated into ambulance management by planning for such events before their occurrence and responding quickly when the need arises.

In the event of an incident, emergency response services require a smart spatial routing decision support system to travel to the incident location in time. This requires supporting the route analysis of emergencies by dispatching appropriate units that are able to respond within a reasonable time frame. For that purpose, there must be a demarcation of EMS service zones, as this will optimize the efficiency of the service facility. Although various strategies have been established to address the problem of spatial partitioning and catchment area demarcation, it remains a challenging task.

The definition of catchment areas for EMS units is one of the most significant pieces of information for emergency management. A catchment is an area within which a particular EMS base is the closest. Voronoi diagrams are a promising technique for this purpose. They are polygons whose borders are within the same distance from the generator points as adjacent polygons [6,7].

In the catchment theory, the closest EMS is typically defined by distance, which is the most common way of determining it. Despite the fact that, in theory, physical distance can be used to measure closeness, this approach does not always work in real practice. Traffic density is constantly changing, making it very difficult to predict catchment areas for time-sensitive applications such as ambulance emergency services. Therefore, shortest-path algorithms cannot rely only on distance but are also highly dependent on traffic as well.

Motivated by this issue, in this study, a smart spatial routing and accessibility analysis system is proposed for EMS using catchment areas of the Voronoi spatial model and time-based Dijkstra’s routing algorithm to support the route analysis of emergencies so that appropriate units can be dispatched and reach their destination within a reasonable time frame. Some improvements to the Dijkstra algorithm have been made, such as the addition of a dynamic traffic factor based on a time function to the Dijkstra algorithm computation called time-based Dijkstra’s routing algorithm (TDRA). In this method, the road distance is estimated by the travel time instead of the shortest distance and takes into account cost parameters such as length, velocity, and saturation flow as edge weights in the traffic travel time.

In summary, the following are the key contributions of this work:

- Catchment areas from the Voronoi spatial model and time-based Dijkstra’s routing algorithm are proposed for EMS to present a smart spatial routing and accessibility analysis system.
- Model evaluations and proof of concepts are conducted using real-world datasets of ambulance locations and road networks in the metropolitan area of Riyadh, Saudi Arabia.

This research paper is organized as follows: Section 2 gives an overview of the research relevant to this study. Section 3 explains the prerequisites concept of the Voronoi diagram. Section 4 explains the proposed system architecture. Section 5 discusses the study area and data. Section 6 discusses the real-world implementation in Riyadh. Section 7 concludes the paper and indicates our proposed future work.

## 2. Related Work

Several studies have explored this problem and have provided several methods that can be generally defined as route optimization. Minimizing the emergency response time requires determining the most efficient optimization techniques and variables to reduce emergency vehicle travel time. Most emergency management systems rely on specialized software to locate, dispatch and route emergency vehicles. Emergency vehicles are outfitted with software to assist drivers in the location of an emergency with specific traffic information.

Such software employs route optimization to ensure that the vehicles reach their destination on time. For example, the time-based or distance-based route may be obtained using Google Maps API. Refs. [8,9] employed Google Maps for the estimation of the fastest route between points which uses a combination of information about the road network. However, the resulting generated polygons do not reflect the network Voronoi diagram (NVD). This is due to the fact that distances are calculated from point to point in the maps API instead of calculating the distances to all potential destinations from a single source.

In addition, the shortest route between any emergency unit and emergency location might be the best way to minimize travel distance. Dijkstra’s shortest path algorithm or A*algorithm are well known in computer science for such optimization, and several of the examined works employed it in some form. Elmandili et al. [10] developed the vehicular ad hoc network (VANET)-based navigation, which also employed Dijkstra’s routing algorithm. Barzegar et al. [11] proposed a spatial experience-based route-finding algorithm using ontology. The route-finding algorithm was implemented using Dijkstra’s algorithm. Nordin et al. [12] proposed the use of the A* algorithm and the road network in the creation of an ambulance routing system. Chen et al. [13] used Dijkstra’s algorithm to calculate the optimal route for various traffic scenarios, such as the morning peak, evening peak, and midday. Brady and Park [14] described a shortest-path algorithm for routing EVs (Emergency Vehicle Specialists) with additional road features, including lane count, intersection control devices, and construction works.

Several studies propose various approaches for emergency vehicle routing based on GIS software technology. In [15], GIS-based network analysis was conducted and applied to the Greater Cairo road network. The purpose of this study was to determine the best route between two locations on the road network and to locate the nearest healthcare service providers based on the time it took to travel to an incident location. The authors implemented the network analysis using the Dijkstra routing algorithm built into the ArcGIS network analyst extension. Dabhade et al. [16] solved the challenge of determining the shortest path to a specialty hospital in Aurangabad, Maharashtra State, India, using ArcGIS software and Dijkstra’s algorithm. The shortest path was determined using road distance. Gubara et al. [17] created a GIS-based healthcare emergency response system to determine the best path from an event location to the nearest healthcare service provider. They determined that the shortest way was the optimal route. Fitro et al. [18] provided a problem-solving strategy in the shortest path search based on GIS by combining the node combination algorithm with Dijkstra’s algorithm.

Derekenaris et al. [19] combined data from GIS, GPS, and GSM technologies in Greece to route ambulances. The ambulance chose the shortest route to the incident location. Kai et al. [2] constructed a GIS-based emergency response system and the shortest path analysis based on the Dijkstra algorithm. Nicoara and Haidu [20], Winn [21], and Sun et al. [22] employed GIS-based networks to discover the shortest path access for emergency vehicles utilizing real-time traffic data. Tlili et al. [23] presented a solution to the ambulance route issue. Shahparvari et al. [24] proposed a vehicle routing problem (VRP) model to improve the efficacy and efficiency of emergency response and rescue operations on short notice. In [25], an emergency response management system based on GIS was developed in Delhi, India. Hsu et al. [26] proposed a decision support system to improve response times in emergency situations during typhoon strikes in Taiwan. Berman et al. [27] presented a methodology to determine the optimal location of specialized response team stations so they could respond within a certain time threshold to hazmat incidents in the region.

Some studies suggest alternative methods for routing emergency vehicles. Chanta et al. [28] explored three bi-objective models to ensure an equitable distribution of EMS ambulances across rural and urban areas. Amr et al. [29] proposed a distributed emergency ambulance (DEA) routing system to reduce the emergency ambulance response times affected by traffic or incorrect human decision-making, which includes routes to guide emergency vehicles and assign distributed emergency resources. Wang and Liu [30] used RFID tags on ambulances and wireless sensor nodes on the roads to collect real-time traffic data and forecast the fastest routes. Drive catchment applications are also often employed in traffic analysis studies [31] and emergency services coverage [32,33,34]. Souza et al. [35] proposed the CIDMERA traffic system, which enhances the overall spatial use of a road network while simultaneously lowering average vehicle travel costs by preventing vehicles from being stopped in traffic.

A survey of the most recent literature reveals that solutions to emergency response systems focus primarily on the shortest distance factor as the main criterion without considering factors affecting travel through the network. However, time-sensitive applications, such as emergency services, do not function effectively due to the lack of timely information. This study proposes a method using catchment areas for emergency services utilizing the NVD approach and time-based Dijkstra’s routing algorithm to present a smart spatial routing and accessibility analysis system. The catchment concept is used to provide a visual representation of the coverage. The catchment areas enable the creation of extremely useful catchment maps, helping the system to perform faster and more effective catchment analysis. Also, the Dijkstra algorithm has been improved by adding a dynamic traffic factor based on a time function called time-based Dijkstra’s routing algorithm (TDRA). Travel time is used in this concept instead of physical distance and cost parameters, such as length, velocity, and saturation flow, which are considered edge weights. This will facilitate the route analysis of emergencies so that a suitable unit can be dispatched and reach the emergency site as quickly as possible.

## 3. Network Voronoi Diagrams

The Voronoi Diagram (VD) is one of the most widely-used mathematical models for simulating catchment areas of multiple facilities and has been intensively applied in spatial databases and computational geometry. The Voronoi diagram shows the division of a particular area into multiple parts called Voronoi cells. Using Voronoi cells, each facility point creates an independent area without overlapping with other facilities [6,7]. The following definitions describe these concepts.

**Definition** **1.** *Voronoi cell C(p) = {n|d(n, p${}_{i}$) ≤ d(n, p${}_{j}$), i ≠ j} refers to the area in which any point n is situated in a Voronoi cell C(p${}_{i}$) and considers p${}_{i}$ to be the nearest facility point.*

**Definition** **2.** *The Voronoi diagram VD of a set of Voronoi cells where: VD = {C(p${}_{1}$), C(p${}_{2}$), ⋯, C(p${}_{n}$)}.*

**Figure 1:**
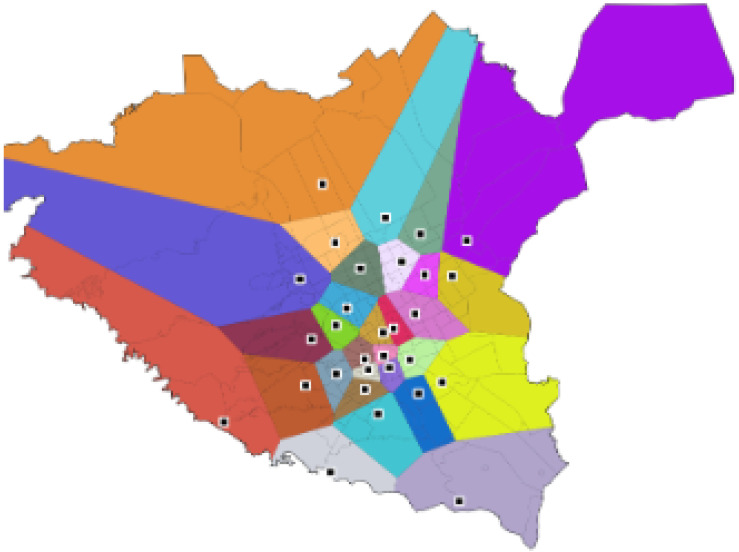
Emergency medical services catchment in Riyadh using Voronoi diagram.

Figure 1 depicts the use of a Voronoi diagram to calculate the catchment area for emergency medical services in Riyadh. Each different color reflects the catchment region for each EMS. A catchment region for an EMS is a Voronoi cell. It indicates the location where the nearest EMS is the generating point for this specific Voronoi cell. Figure 1 illustrates a Voronoi diagram without taking into account the road infrastructure.

**Figure 2:**
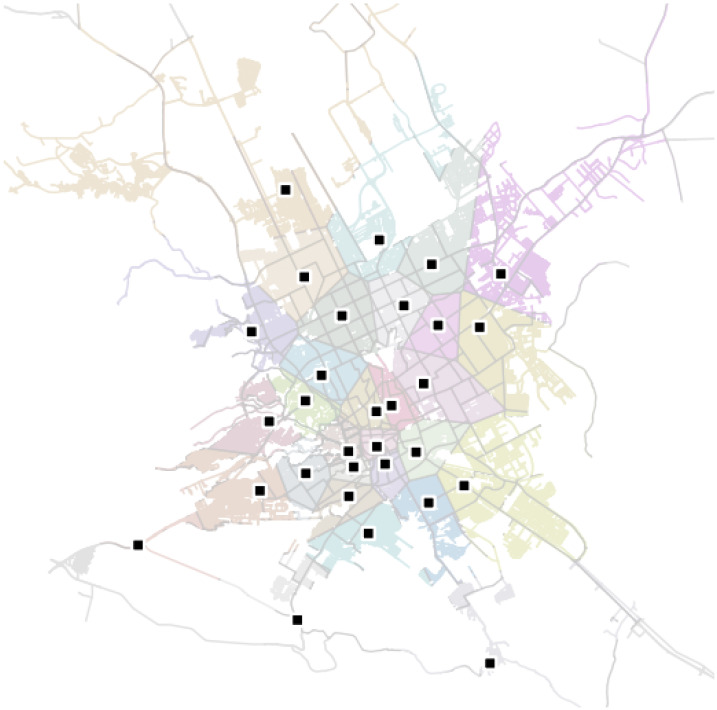
Road infrastructure catchment using NVD.

An example of a more realistic driving catchment area can be seen in Figure 2, where the catchment is estimated while taking road infrastructure into account using network Voronoi diagrams (NVD). Network Voronoi diagrams (NVD) differ from Voronoi diagrams (VD) in that the distance between two points in NVD is the shortest path instead of the Euclidean distance [36,37,38].

**Definition** **3.** *P = {p${}_{i}$, ⋯, pj} is a set of generators that are considered the set of POIs for a road network, and N = {n${}_{i}$, ⋯, n${}_{j}$} is the node-set. Every node within the Voronoi polygon of p${}_{i}$ is always closer to p${}_{i}$ than other POIs for every p${}_{i}$∈ P.*

## 4. A Smart Spatial Routing and Accessibility Analysis System for EMS

**Figure 3:**
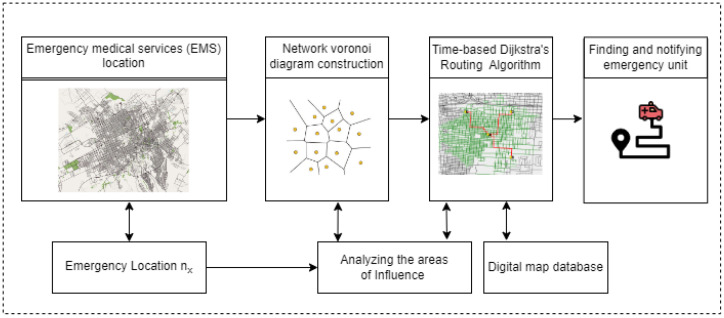
Proposed system architecture.

In ambulance management, an incorrect dispatch can result in a life-and-death situation if the ambulance unit does not arrive on time. A smart spatial routing and accessibility analysis system is proposed for EMS to assist in the routing analysis of emergencies in order to dispatch appropriate units within a maximum response time for emergency situations. Figure 3 shows the proposed system architecture.

In order to model the constraints of a road network for emergency medical services, a road network is originally modeled as an undirected graph, which is a collection of edges and nodes as follows:

**Definition** **4.** *A road network is formalized as G = (N, L) and as a weighted undirected graph where*

- *N = {n${}_{1}$, ⋯ , n${}_{i}$} is the set of nodes of G that includes the POI set P = {p${}_{1}$, ⋯ , p${}_{j}$},*
- *L = {l${}_{1}$, ⋯ , l${}_{m}$} is the set of links (edges) that represent the road segments.*

**Figure 4:**
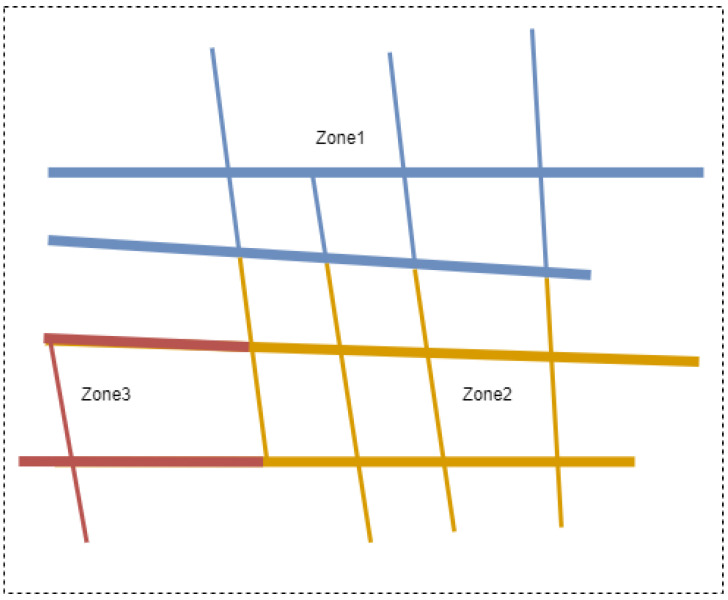
A spatial Voronoi diagram showing the road network for three emergency zones.

First, network Voronoi diagrams (NVD) was generated for the set of POIs (i.e., EMS) *P* = {*p*${}_{1}$, ⋯, *p*${}_{j}$}. Voronoi diagrams divide spatial networks (road networks crossed by vehicles) into Voronoi subnetworks based on discrete points called Voronoi generators. Each Voronoi generator (EMS zone) creates a sub-network that comprises all the points that are closer to this generator than the others (see Figure 4).

Let *R*(*N*, *L*) be a network of nodes $N=\{n_{1},n_{2},\cdots ,n_{i}\}$ and link $L=\{l_{1},l_{2},\cdots ,l_{m}\}$. The EMS unit zones are modeled by Voronoi generators $P=\{p_{1},p_{2},\cdots ,p_{j}\}$ with *P* ⊂ *N*. *n* (emergency location) can be, and *p* (emergency unit) can be considered as two nodes belonging to the set N. For the point, *n* and *p* on the link in *L*, *dis*(*n*, *p*) is defined as the shortest distance using the road network between point *n* and point *p*.

**Definition** **5.** (Adjacent POIs). *Assume the nearest POIs (EMS) of a node n are p${}_{1}$, ⋯, p${}_{j}$, then pj is in the adjacent POIs of p${}_{1}$, ⋯, p${}_{j-1}$.*

**Definition** **6.** *n${}_{i}$ is the demand node i ∈ I that is served by an ambulance p${}_{j}$, which is defined as*

*p${}_{j}$= $\left\{\begin{array}{cc} 1 & if\;an\;ambulance\;is\;located\;at\;the\;candidate\;catchment\;area\;j\in J;\\ 0 & if\;not,find\;the\;p\;new\;EMS\;in\;the\;adjacent\;catchment\;area\;that\;has\;the\;shortest\;travel\;time. \end{array}\right.$*

Based on NVD, a four-phase selection process is implemented to determine the best ambulance unit for responding to the demand node $n_{i}$. The first phase determines the catchment area of the request. The second phase generates candidate units, which contain all the possible POIs (EMS units) within the catchment areas of the Voronoi spatial model and the adjacent regions. In the third phase, the distances from $n_{i}$ to the candidate POIs are calculated and selected only after calculating the shortest distance based on the time function. After determining the nearest POI within the catchment area, the proposed system then iterates j−1 times to search for the remaining j−1 nearest the POIs of a node on the basis of the adjacent relationships among vertices according to Definition 5.

**Figure 5:**
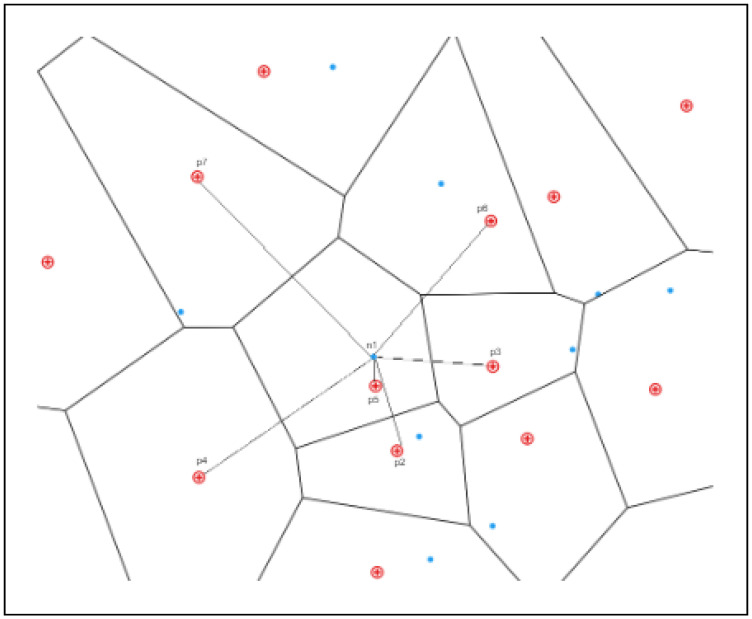
Example of the nearest POIs (EMS) from $n_{1}$.

For example, in Figure 5, we intended to search for the nearest POIs from $n_{1}$ (the emergency location). It is obvious to see that p5 is the candidate’s nearest POI (i.e., EMS) based on distance in terms of travel time. When the EMS unit $p_{j}$ = 0 (Definition 6), then we can obtain the candidate set adjacent to POI as {$p_{3}$, $p_{6}$, $p_{7}$, $p_{4}$, $p_{2}$}. Then, we can compute the distances (shortest path time) between $n_{1}$ and each POI, i.e., *dis*($n_{1}$, $p_{3}$), *dis*($n_{1}$, $p_{2}$), *dis*($n_{1}$, $p_{6}$), *dis*($n_{1}$, $p_{4}$), and *dis*($n_{1}$, $p_{7}$). Finally, we compared the paths and chose the shortest among them; for example, let us say *dis*($n_{1}$, $p_{3}$) has the shortest travel time.

Based on the graph search, the node, and link (edge), Dijkstra’s algorithm can determine the shortest path (i.e., the path with the lowest cost) between a given source node and every other node in the graph. Dijkstra’s original algorithm calculates the shortest paths based only on the distances between the nodes [39,40]. However, it cannot be applied to a traffic-dependent network. When traffic depends on a network, the shortest path algorithms cannot depend only on distance but will be highly dependent on traffic as well. Some improvements to the Dijkstra algorithm have been developed, such as the addition of a dynamic traffic factor based on a time function to the Dijkstra algorithm computation called time-based Dijkstra’s routing algorithm (TDRA).

Travel time is defined as the time spent traveling on the road segment. The travel time generally comprises the running time and delay time. The TDRA concept is based on evaluating distances in terms of travel time rather than the actual distance. This is because the actual distance does not always accurately reflect the distance between two points, particularly in densely populated areas and in time-critical applications such as those involving emergency services. The steps applied for the estimation of travel time for a segment of road are presented below.

It can be assumed that *l* ($n_{x}$, $n_{y}$) is the road segment connecting nodes $n_{x}$ and $n_{y}$ with $dl_{n_{x}}$,${}_{n_{y}}$ > 0 representing the distance of the road segment *l*. The allowable velocity limit of the road segment is denoted as $s_{l}$. $s_{l}$ is the maximum permitted speed of the road segment *l*. For safety reasons, speed limits should be adapted to prevailing road conditions. To regulate traffic speed, low-speed roads typically feature speed-limiting obstacles such as speed humps or uneven surfaces. Therefore, speeding is considered dangerous when driving above the speed limit. When a road segment is clear of traffic jams, the time limit $tl^{free}$ is the time it takes to travel from $n_{x}$ to $n_{y}$ at the constant speed of $s_{l}$. As a result, $s_{l}$ is a realistic limit where the real vehicle speed corresponds to the speed restriction on the road. The following definition formalizes the concept of $tl^{free}$:

**Definition** **7.** (Time limit $tl^{free}$). *Time limit tl${}^{free}$ (second) can be formulated as follows:*

*tl${}^{free}$= $\frac{\frac{d_{l}}{1000}}{s_{l}}\times \left(3600\right)$ where*

- *d${}_{l}$ represents distance (meters) for road segment l;*
- *s${}_{l}$ represents allowable velocity limit.*

It is worth noting that $d_{l}$ and $s_{l}$ are static values that can be obtained from the digital map database. If a road segment has traffic congestion, $k_{l}$ the present traffic density (i.e., the number of vehicles per km) of l is used to estimate the unexpected delay when traffic jams occur.

**Definition** **8.** (Traffic density). *The traffic density on a specific road segment l is calculated as: k${}_{l}$ = $\frac{n\times 1000}{d_{l}}$ where*

- *n identifies the no. of vehicles occupying a length d${}_{l}$ of the road;*
- *d${}_{l}$ represents distance (meters) for road segment l.*

**Definition** **9.** *Saturation flow describes the maximum traffic flow as:*

*$\frac{k_{l}}{k_{l}^{max}}$ = $\frac{current\;traffic\;density\;of\;vehicles\;on\;l}{maximum\;traffic\;density\;of\;vehicles\;on\;l}$.*

**Definition** **10.** (Minimal travel time). *The Minimal travel time over the road segment l can be formulated as: tl${}^{min}$= $\frac{\frac{d_{l}}{1000}}{s_{l}\times \left(1-\frac{k_{l}}{k_{l}^{max}}\right)}\times \left(3600\right)$.*

TDRA construction uses a weighted graph to represent the structure of road networks, similar to NVD and uses the multi-source Dijkstra algorithm. The main difference is that the proposed TDRA construction combines all cost parameters (length, velocity, saturation flow) as edge weights in traffic travel time instead of physical distance (length). The smart spatial routing and accessibility analysis system can be summarized as the pseudo-code shown in Algorithm 1.
**Algorithm 1:** Pseudo-code algorithm of the proposed system.
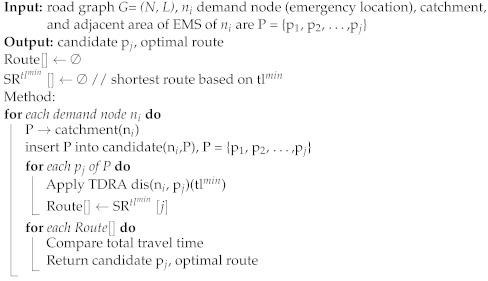


## 5. Data and Study Area

**Figure 6:**
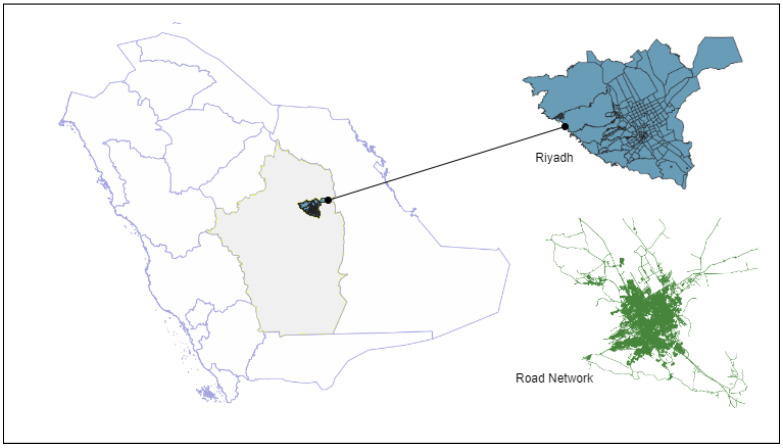
Study area.

This research was carried out in the metropolitan region of Riyadh, Saudi Arabia. Riyadh is the capital of Saudi Arabia and the largest city on the Arabian Peninsula (Figure 6). It has a total population of ∼7,388,000 people. The city is located 600 m above sea level [41]. The city area is 5400 km${}^{2}$. According to the Riyadh High Commission for Development, the area of urban settlements is 1298 km${}^{2}$, including the road network area. The city’s administrative boundaries are split into 18 municipalities and 209 neighborhoods [42]. As the country’s capital, the area is important for housing and industry as well as for the country’s economy.

**Figure 7:**
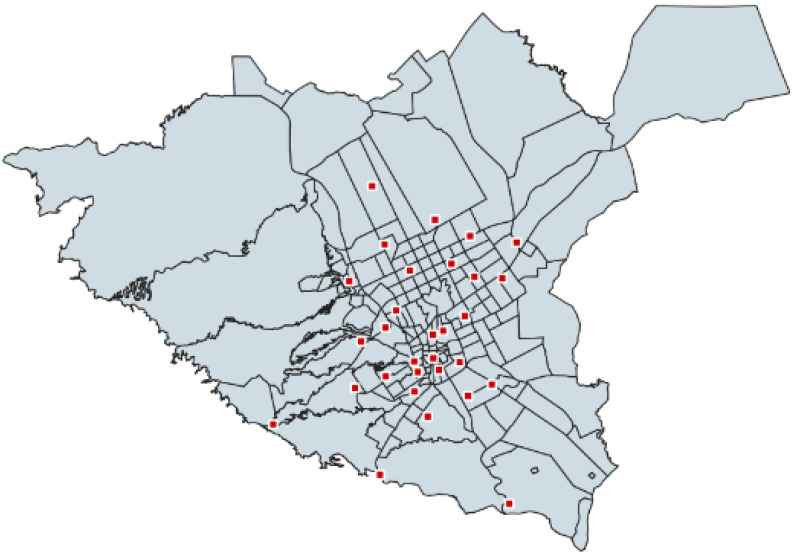
Locations of 30 EMS ambulance unit stations in Riyadh.

This study utilized data obtained from EMS ambulance stations and road networks. The EMS ambulance station dataset includes information such as name, coordinate position data of ambulance units (latitude, longitude), and district. Figure 7 shows the positions of 30 EMS ambulance unit stations. The road network dataset provided highways and local roads in Riyadh city. The speed limit information in this dataset was utilized to perform a route-based analysis. For routes with no speed limit information, the computed average route speed of 40 km/h was utilized as the travel speed.

## 6. Results and Analysis

The experimental results of the simulation for the proposed smart spatial routing and accessibility analysis system for EMS are presented in this section. The simulations were run in a 64-bit Windows 10 environment with an Intel Core i7 processor with 16 GB of system RAM. The dataset was collected utilizing the crowdsourcing map platform OpenStreetMap (OSM). The dataset includes road network data from OSM and ambulance station data in Riyadh city. The road trajectory data were preprocessed to remove all noise and anomalies prior to the evaluation process. The datasets are all locally stored. The simulation was built using “Python” and the “PostgreSQL/PostGIS database management system", which can process large amounts of spatial data.

In this simulation experiment, a set of random addresses were generated for catchment areas to compute a minimal time limit between the address and the nearest emergency POI. By calculating the cost of each road segment, the shortest path between the starting and destination points was determined using TDRA. Furthermore, the saturation level involved in this simulation experiment was considered to be 0.70. The saturated flow level indicator has a value between 0 and 1. In general, a saturation level below 0.70 indicates that the capacity is still sufficient for the amount of traffic. However, once the saturation level reaches one, the traffic flows can become erratic, causing congestion and delays. In the route analysis, the value of the saturation flow is a constant based on user-entered parameters, but in reality, this value constantly changes from time to time. Consequently, experiments will be carried out in the future to develop this architecture for practical use.

**Figure 8:**
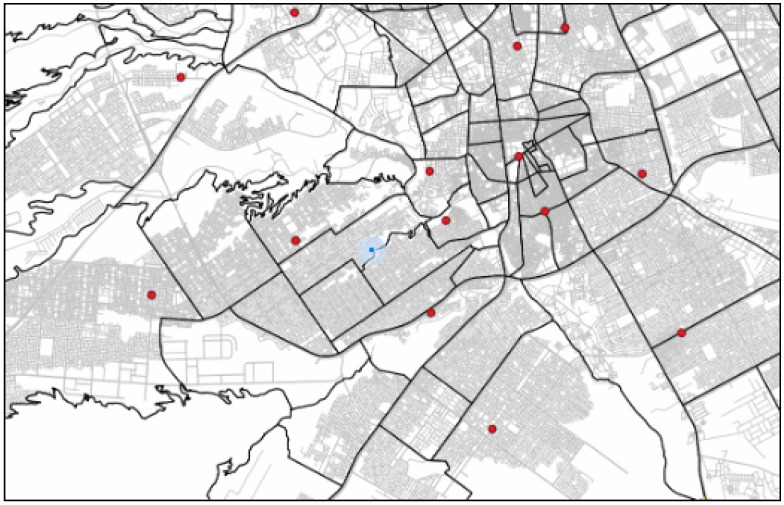
Catchment area of the emergency locations.

**Figure 9:**
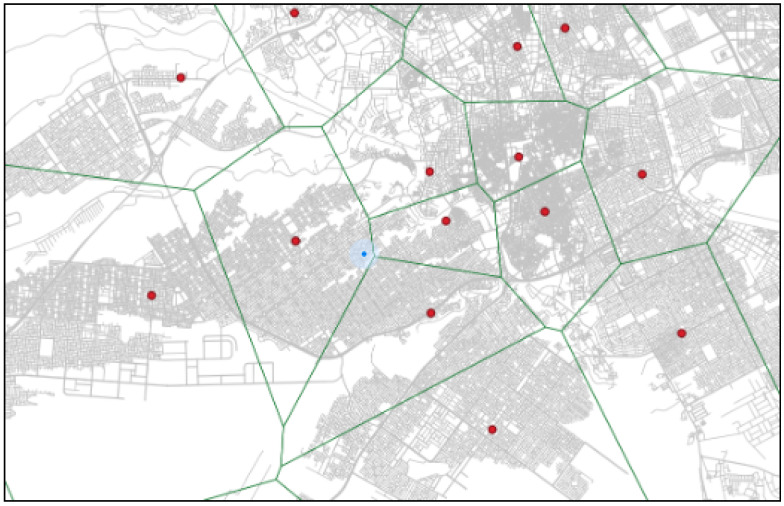
Catchment areas of Voronoi spatial model.

The system was tested by simulating an emergency scenario between the demand node, ’the emergency incident location’, and the EMS ambulance station node (Figure 8). First, the system reads the coordinate point of the emergency location. After the system reads the input, and the catchment areas of the emergency locations are determined in the map system (Figure 9). The ambulance emergency units are then selected within the catchment area and in the adjacent areas of the Voronoi spatial model.

**Figure 10:**
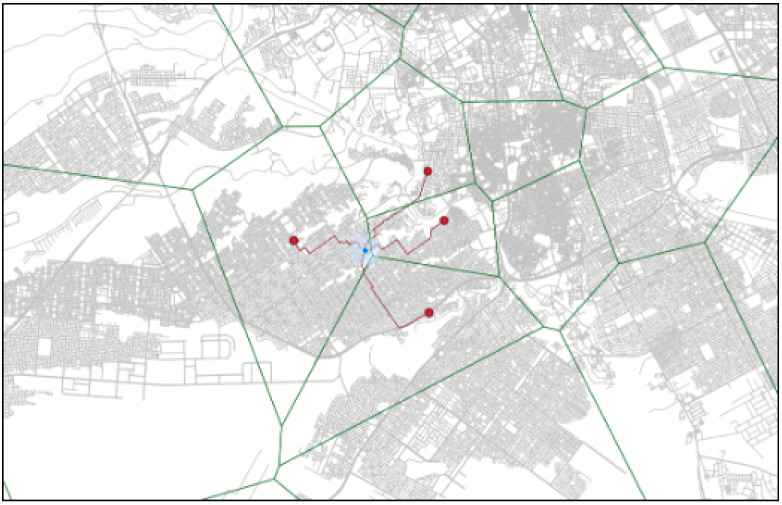
Candidate ambulance units within the catchment areas.

**Figure 11:**
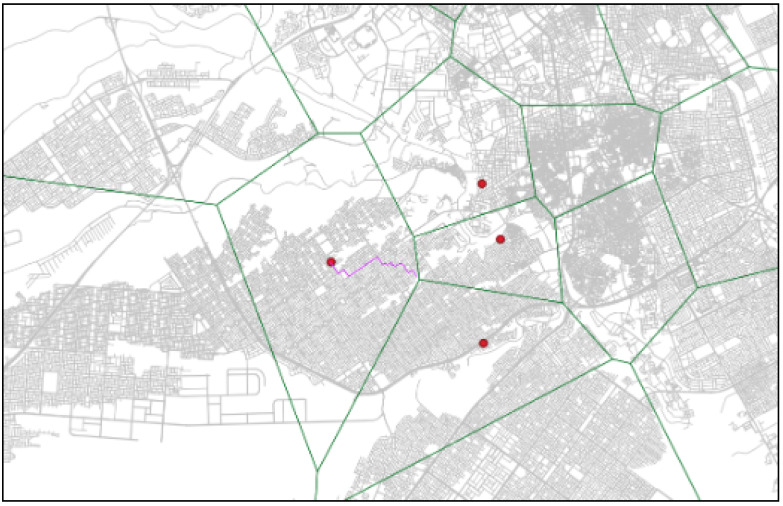
Ambulance emergency unit that has the minimum travel time.

From the simulation of this emergency incident, the model successfully determined the nearest candidate ambulance unit within the catchment area and three candidates EMSs in the adjacent catchment areas that had the minimum travel time to the demand point, considering TDRA construction. The four candidate ambulance units for this emergency scenario can be seen in Figure 10. Figure 11 shows the ambulance emergency unit that is selected as the candidate point based on the minimum travel time.

**Figure 12:**
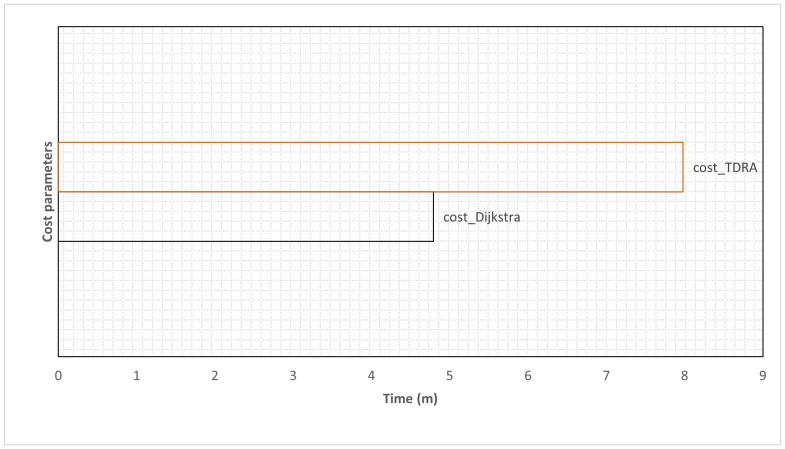
Impact of the cost parameters on the total travel time with Dijkstra’s algorithm.

Also, an experiment was conducted to evaluate the trajectory cost between the demand points and the emergency points. The route options show the total travel time from the values of cost_Dijkstra and cost_TDRA as shown in Table 1. The results illustrate the difference in travel time between the two cost values. The proposed TDRA construction combines all cost parameters (length, velocity, saturation flow) to calculate the optimal route to the demand point. Figure 12 shows the effect of the cost parameters on the total travel time with Dijkstra’s algorithm. In reality, in high-density regions and for time-critical applications such as emergency services, physical length is not always an accurate measure of travel time between two particular points.

Discussion: Unlike route computation, which is usually based on cost minimization, either the classical Dijkstra algorithm or the A* algorithm is usually employed for this computation; the proposed system provides an effective service area division of EMS stations through catchment areas of the Voronoi spatial model. The catchment concept is applied to offer a visual representation of coverage. Catchment areas enable the creation of extremely useful catchment maps, helping the system to perform faster, and providing more effective catchment analysis. Also, the Dijkstra algorithm has been improved by adding a dynamic traffic factor based on a time function called time-based Dijkstra’s routing algorithm (TDRA). The cost value calculation using the TDRA construction algorithm is combined using various parameters to evaluate the maximum routing for EMS to the emergency location. Travel time is used in this concept instead of physical distance and cost parameters, such as length, velocity, and saturation flow, which are considered edge weights. Based on system experiments, it is found that the search for optimal routing by employing the proposed system algorithm can assist EMS in calculating the arrival time at the scene of an emergency. Considering road conditions and obstacles, with the travel time as the cost, we plan to conduct experiments in the future to measure the accuracy of determining the travel time.

## 7. Conclusions

This paper proposed a catchment area for emergency services utilizing the NVD approach and time-based Dijkstra’s routing algorithm to present a smart spatial routing and accessibility analysis system. This had the aim of facilitating the route analysis of emergencies so that a suitable unit could be dispatched to reach the emergency sites as quickly as possible. The Dijkstra algorithm has been improved by adding a dynamic traffic factor based on a time function called time-based Dijkstra’s routing algorithm (TDRA). Travel time is used in this concept instead of physical distance and cost parameters such as length, velocity, and saturation flow, which are considered edge weights.

In future work, this system will be improved to estimate travel time by taking into consideration road conditions and obstacles. Also, future experiments will be conducted so that this system can be developed for practical applications.

## Figures and Tables

**Table 1:** Comparison total travel time considering the cost parameters with Dijkstra’s algorithm.

Cost	Parameter	Expected Total Travel Time
Cost_Dijkstra	length	4.79 m
Cost_TDRA	length, velocity, saturation flow	7.98 m

## Data Availability

The experimental data are contained within the article.
